# Did you see it?

**DOI:** 10.7554/eLife.107246

**Published:** 2025-05-28

**Authors:** Ling Liu

**Affiliations:** 1 Cognitive Science and Allied Health School, Beijing Language and Culture University Beijing China; 2 Speech and Hearing Impairment and Brain Computer Interface LAB, Beijing Language and Culture University Beijing China

**Keywords:** consciousness, neural measures of consciousness, subjective measures, signal detection theory, criterion, bias, Human

## Abstract

Cautious reporting choices can artificially enhance how well analyses of brain activity reflect conscious and unconscious experiences, making distinguishing between the two more challenging.

**Related research article** Fahrenfort JJ, Johnson PA, Kloosterman NA, Stein T, van Gaal S. 2024. Criterion placement threatens the construct validity of neural measures of consciousness. *eLife* **13**:RP102335. doi: 10.7554/eLife.102335.

Have you ever considered how your brain determines when you have clearly seen something and when you have not? It is possible to be shown something, such as an image, that our brain registers even if we are not consciously aware that we have seen it. Researchers have developed diverse theories that seek to explain these mechanisms of consciousness and unconsciousness, as well as the distinctions between them (Mudrik et al., 2025; Yaron et al., 2022).

Investigating this often involves recording people’s brain activity while they are shown stimuli at various perception levels and asked to report whether they have seen them or not. The recordings are then analysed to identify brain activity patterns associated with unconscious or conscious perception through a process known as “neural decoding”. However, some participants may report having seen a stimulus only when they are absolutely certain they have detected it, while others may report it even if they are not fully confident. This highly subjective threshold is known as the judgement criterion (Green and Swets, 1966). As it varies among individuals, it can introduce bias into experimental data, which often complicates studies of conscious experiences. Now, in eLife, Johannes Fahrenfort (Free University of Amsterdam) and colleagues in Leiden, Hamburg and Amsterdam report that differences in the judgment criterion have distinct effects on how neural signals associated with conscious and unconscious experiences are interpreted (Fahrenfort et al., 2024).

Fahrenfort et al. began by using a simple computational model to simulate how neural decoding might be influenced by differences in the judgement criterion. The team simulated two different scenarios. In the liberal condition, participants reported seeing a target stimulus even when the signal was weak or absent. On the other hand, in the conservative condition participants only reported seeing the stimulus when the signal was strong, causing them to report “seeing” it less often (Figure 1A). Analysis showed that the conservative condition led to higher neural decoding performance than the liberal condition, with brain activity patterns associated with a target stimulus being present or not more easily distinguished (Figure 1B). This apparent enhancement in neural decoding accuracy is a result of the conservative reporting itself rather than any change in the stimulus, which remained the same across the different conditions.

**Figure 1.**
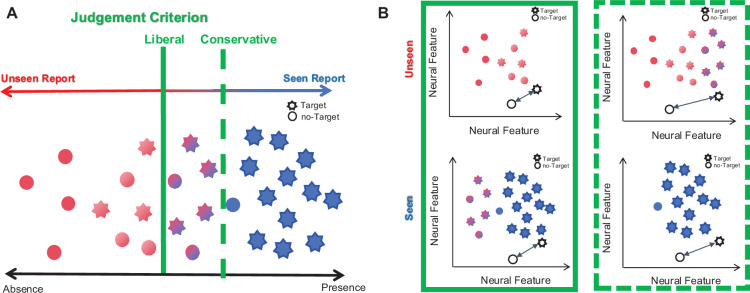
A conservative judgement criterion improves neural decoding performance in experiments aimed at discriminating between conscious and unconscious experiences. (**A**) To identify differences in the brain activity associated with conscious and unconscious experiences, researchers measure participants’ brain activity as they report whether they have seen (red) or not seen (blue) a visual stimulus. These reports are influenced by an individuals’ judgement criterion, which reflects how readily they will report seeing a stimulus even if they are not entirely sure they did. The stimuli range in perceptual clarity from not eliciting a conscious experience (absence) to being very clearly visible (presence). A liberal criterion (solid green line) means that participants more readily report seeing a stimulus, even if it is weak or absent. This results in higher rates of accurate stimulus reporting (red stars), as well as false alarms (red circles). In conditions where the judgement criterion is conservative (green dashed line), participants only report strong signals as “seen”, resulting in lower rates of target stimulus reporting (blue stars) and false alarms (blue circles). Thus, the judgment criterion does not alter the experience but only influences how it is conveyed. For example, those in the liberal condition would call the purple shapes “seen”, whereas those in the conservative condition would report them as “unseen”. (**B**) Analysis by Fahrenfort et al. showed that these reporting strategies lead to differences in neural decoding performance. When the conservative criterion (dashed green line box) is applied, neural features from both “seen” and “unseen” trials become more distinctly clustered i.e. there is a greater distance between the target (star) and non-target brain patterns (circle) (depicted by the length of the arrows), leading to improved decoding performance when compared with the liberal criterion (solid green line box). This enhanced performance does not reflect more accurate perception but just a tendency to only report strong visual stimuli, which complicates interpretation of conscious and unconscious experiences.

To validate these findings, Fahrenfort et al. next compared the simulated results with two comparable experimental datasets. In the first dataset (which was reported by Kloosterman et al., 2019; Kloosterman et al., 2020), the experimental model matched the one used in the simulated experiments, with participants reporting on whether a visual stimulus was either "seen" or "unseen". In the second dataset, which was collected by Fahrenfort et al., participants used the Perceptual Awareness Scale (PAS; see Ramsøy and Overgaard, 2004) to report how clearly they saw the visual stimulus, with 0 indicating no conscious experience (similar to "unseen"), while 1, 2, and 3 represented the visual stimulus being increasingly clearer to see. Using this scale should make reporting more precise, as a value of 0 should only be given when the stimulus is truly “unseen”. In both experiments, researchers used feedback to shift participants' judgment criterion, successfully prompting them to respond more liberally or conservatively during the task. Fahrenfort et al. analyzed how neural decoding performance was influenced by these shifts in the judgement criterion. As in the simulations, adopting a conservative approach to reporting led to increased neural decoding performance. However, this effect was only observed in the “unseen” condition in the first dataset and the “seen” condition in the second dataset.

To better understand this inconsistency, Fahrenfort et al. used further computational modelling to show that neural decoding performance is not only affected by the judgement criterion but also by a participant’s signal sensitivity, which is how well they are able to detect a stimulus regardless of their judgement criterion. This can be measured by comparing how often a participant correctly detects a visual stimulus with how often they mistakenly report seeing one, revealing their actual perceptual ability. When participant sensitivity was low, a more conservative judgement criterion significantly inflated the neural decoding performance in the “seen” condition. However, when participant sensitivity was high, a conservative bias significantly inflated the neural decoding accuracy under the “unseen” condition (Figure 1B).

Taken together, these findings confirm the long-held prediction that subjective reporting biases can influence how well conscious and unconscious experiences can be distinguished from one another experimentally. This highlights the importance of carefully controlling the effects of the judgement criterion in future studies.
